# Lifetime effects and cost-effectiveness of statin therapy for older people in the United Kingdom: a modelling study

**DOI:** 10.1136/heartjnl-2024-324052

**Published:** 2024-09-10

**Authors:** Borislava Mihaylova, Runguo Wu, Junwen Zhou, Claire Williams, Iryna Schlackow, Jonathan Emberson, Christina Reith, Anthony Keech, John Robson, Richard Parnell, Jane Armitage, Alastair Gray, John Simes, Colin Baigent, Cholesterol Treatment Trialists’ Collaborators, J Armitage

**Affiliations:** 1 Health Economics Research Centre, Nuffield Department of Population Health, University of Oxford, Oxford, UK; 2 Health Economics and Policy Research Unit, Wolfson Institute of Population Health, Queen Mary University of London, London, UK; 3 Clinical Trial Service Unit and Epidemiological Studies Unit, Nuffield Department of Population Health, University of Oxford, Oxford, UK; 4 NHMRC Clinical Trials Centre, The University of Sydney, Sydney, New South Wales, Australia; 5 Clinical Effectiveness Group, Wolfson Institute of Population Health, Queen Mary University of London, London, UK; 6 Patient and Public Representative, Havant, UK

**Keywords:** Health Care Economics and Organizations, Computer Simulation, Cardiovascular Diseases, Outcome Assessment, Health Care

## Abstract

**ABSTRACT:**

**Background:**

Cardiovascular disease (CVD) risk increases with age. Statins reduce cardiovascular risk but their effects are less certain at older ages. We assessed the long-term effects and cost-effectiveness of statin therapy for older people in the contemporary UK population using a recent meta-analysis of randomised evidence of statin effects in older people and a new validated CVD model.

**Methods:**

The performance of the CVD microsimulation model, developed using the Cholesterol Treatment Trialists’ Collaboration (CTTC) and UK Biobank cohort, was assessed among participants ≥70 years old at (re)surveys in UK Biobank and the Whitehall II studies. The model projected participants’ cardiovascular risks, survival, quality-adjusted life years (QALYs) and healthcare costs (2021 UK£) with and without lifetime standard (35%–45% low-density lipoprotein cholesterol reduction) or higher intensity (≥45% reduction) statin therapy. CTTC individual participant data and other meta-analyses informed statins’ effects on cardiovascular risks, incident diabetes, myopathy and rhabdomyolysis. Sensitivity of findings to smaller CVD risk reductions and to hypothetical further adverse effects with statins were assessed.

**Results:**

In categories of men and women ≥70 years old without (15,019) and with (5,103) prior CVD, lifetime use of a standard statin increased QALYs by 0.24–0.70 and a higher intensity statin by a further 0.04–0.13 QALYs per person. Statin therapies were cost-effective with an incremental cost per QALY gained below £3502/QALY for standard and below £11778/QALY for higher intensity therapy and with high probability of being cost-effective. In sensitivity analyses, statins remained cost-effective although with larger uncertainty in cost-effectiveness among older people without prior CVD.

**Conclusions:**

Based on current evidence for the effects of statin therapy and modelling analysis, statin therapy improved health outcomes cost-effectively for men and women ≥70 years old.

**WHAT IS ALREADY KNOWN ON THIS TOPIC**

- Randomised studies showed that statins reduce the incidence of myocardial infarction and ischaemic stroke by about one quarter for every 1 mmol/L reduction in low-density lipoprotein cholesterol but direct evidence among older people without prior cardiovascular disease (CVD) is limited.
- In previous studies, statin therapy has been shown to be cost-effective in older people, but it has been suggested that a small further adverse effect would offset its cardiovascular benefit.
- Despite markedly increased CVD risks with advancing age, lower statin use is reported among older people.

**WHAT THIS STUDY ADDS**

- The value of statin therapy was reassessed using a contemporary UK CVD model validated in older people together with the synthesised evidence of statins’ beneficial effects on CVD events and adverse effects on myopathy, rhabdomyolysis and incident diabetes.
- The study reported that both standard and higher intensity statin therapies enhanced health outcomes, with higher intensity therapy achieving larger benefits, and were cost-effective in people ≥70 years old in the UK. These findings remained robust in scenarios with smaller CVD risk reductions and further hypothetical adverse effects with statin therapy, though with increased uncertainty among older people without CVD.

**HOW THIS STUDY MIGHT AFFECT RESEARCH, PRACTICE OR POLICY**

- While ongoing statin trials in older people without CVD will add valuable data, particularly in those over the age of 75 years, statin treatment of individuals should not be delayed while awaiting their findings.
- Increasing statin uptake and adherence among older people will reduce CVD risks.

## Introduction

 Statins are widely available generically and a cornerstone in cardiovascular disease (CVD) prevention. High-quality randomised evidence has shown that statins reduce the incidence of myocardial infarction (MI) and ischaemic stroke by about one quarter for every 1 mmol/L reduction in low-density lipoprotein cholesterol (LDL-C). More intensive statin regimens achieve larger reductions in LDL-C and prevent more atherosclerotic cardiovascular events.^1^ However, there is less definitive evidence for statin benefit among older patients without CVD history^2^ and guidelines stop short of making specific recommendations on initiating statins for primary CVD prevention in older people.^3 4^ Despite the growing proportion of older people (people ≥70 years old make up about 30% of those over the age of 40 years in the UK) and the markedly higher cardiovascular risk with increasing age, lower statin use is reported.^5 6^

Evidence for treatments’ long-term effects and cost-effectiveness guides healthcare decisions in many countries and healthcare systems, including in the UK. Such evidence ensures that by implementing cost-effective treatments, healthcare systems efficiently use their resources to maximise population health. Previous evidence has indicated that statin therapy is likely to be cost-effective for older people, but the estimates were sensitive to further adverse effects of statins or lower statin effectiveness.^7^,^9^ A recent individual participant data meta-analysis of large statin trials strengthened the evidence for efficacy and safety of statins in older people.^2^ Therefore, we set out to reassess the lifetime effects and cost-effectiveness of statin therapy in people ≥70 years old in the contemporary UK population, in categories by prior CVD, sex and LDL-C level, using this evidence^2^ and a new UK CVD microsimulation model.^10^

## Methods

### Study population

The lifetime effects and cost-effectiveness of statin therapy were assessed in categories of UK adults ≥70 years old in the UK Biobank and the Whitehall II cohort studies. All UK Biobank participants ≥70 years old at recruitment into the study (2006–2010), and those who reached this age by subsequent resurveys, were included in the present study from their earliest eligible attendance. All Whitehall II participants ≥70 years old at phase 9 (2007–2009) in Whitehall II were also included. Information on the derivation of participants’ baseline characteristics is presented in the online supplemental methods. To assess the lifetime effects of statin therapy, a model is required that reliably projects individual participant’s morbidity, mortality, quality of life (QoL) and healthcare costs over their lifetimes without and with statin therapy.

### CVD microsimulation model

The CVD microsimulation model has been reported elsewhere.^10^ Briefly, the model was developed using the individual participant data of large statin clinical trials, and calibrated using the UK Biobank’s participant data. The model employs a broad range of socio-demographic and clinical characteristics to project annually the first occurrence of MI, stroke, coronary revascularisation, vascular death, incident diabetes, incident cancer and non-vascular death. Participant characteristics and incident events determined health-related QoL^10^ and primary care and hospital admission costs^11^ in the model. The model was validated in UK Biobank and Whitehall II studies and against national data.

### CVD microsimulation model validation in older people

In the present study, the model performance was further assessed among participants ≥70 years old during follow-up in the UK Biobank and Whitehall II studies using their linked electronic hospital admissions, primary care records (UK Biobank only), cancer registrations and death records to identify MIs, strokes, coronary revascularisations (UK Biobank only), incident diabetes (UK Biobank only), cancers and deaths during follow-up.

### Effects and costs of statin therapy

The Cholesterol Treatment Trialists’ Collaboration (CTTC) individual participant data meta-analysis of large randomised statin trials informed the relative reductions in the risks of cardiovascular events per 1 mmol/L in LDL-C with statin therapy (table 1) of 24% in MI risk, 16% in stroke, 25% in coronary revascularisation and 12% in cardiovascular death.^2^ We assessed the effects of standard (eg, achieving 35%–45% LDL-C reduction: atorvastatin 20 mg/day, rosuvastatin 5–10 mg/day or simvastatin 40–80 mg/day) and higher intensity statin therapy (eg, achieving ≥45% LDL-C reduction: atorvastatin 40–80 mg/day, rosuvastatin 20–40 mg/day) (online supplemental table 1).^12^ The reduction in LDL-C achieved with each level of statin intensity was derived using the therapy’s proportional reduction and participant’s untreated LDL-C level (with the effects of any ongoing statin therapy removed). Meta-analyses of statin therapies informed 9% excess odds of new-onset diabetes with standard^13^ and further 12% excess odds with higher intensity^14^ statin therapy. An overview of cohort studies informed excess rates of myopathy (11 cases per 100 000 treated per year) and rhabdomyolysis (3.4 cases per 100 000 treated per year; 10% case fatality) with statin therapy^15^; with myopathy and rhabdomyolysis effects on QoL informed from a modelling study.^16^ Generic statin medication costs,^17^ costs of consultations^18^ and blood lipids tests^19^ for initiation and monitoring of statin prescribing in the UK National Health Service were included (table 1).

**Table 1:** Statin treatment effects and statin treatment costs

Item	Value	Source
**LDL cholesterol reductions with statin therapy:**		
With standard statin therapy (eg, atorvastatin 20 mg/day, rosuvastatin 5–10 mg/day or simvastatin 40–80 mg/day)	37%–43%; 43% used	Meta-analysis of randomised controlled trials^12^
With higher intensity statin therapy (eg, atorvastatin 40–80 mg/day, rosuvastatin 20–80 mg/day)	48%–58%; 55% used	Meta-analysis of randomised controlled trials^12^
**Effects of statin therapy on cardiovascular events per1 mmol/Lreduction in LDL cholesterol, Risk Ratio (RR) (95%CI)**		Cholesterol Treatment Trialists’ Collaboration individual participant data meta-analysis^2^
Myocardial infarction (MI)	RR 0.76 (0.73 to 0.79)	
Stroke	RR 0.84 (0.80 to 0.89)	
Coronary revascularisation (CRV)	RR 0.75 (0.73 to 0.78)	
Cardiovascular death	RR 0.88 (0.85 to 0.91)	
**Adverse effects of statin therapy on:**		
Incident diabetes, OR (95% CI)		
With standard statin therapy compared with no statin treatment	OR 1.09 (1.02 to 1.17)	Meta-analyses of randomised controlled trials^13^
With higher intensity statin therapy compared with standard statin therapy	OR 1.12 (1.04 to 1.22)	Meta-analyses of randomised controlled trials^14^
Myopathy		
Excess rate per 100 000 treated with statin therapy pre-year (95% CI)	11 (4 to 27)	Overview of cohort studies^15^
Occurrence of myopathy is associated with reduction in QoL over 30 days recovery period. Statin treatment is stopped.	0.017 QALY reduction in year of event	Modelling study.^16^
Rhabdomyolysis		
Excess rate per 100 000 treated with statin therapy per year (95% CI)	3.4 (1.6 to 6.5)	Overview of cohort studies^15^
Case fatality	10%	Overview of cohort studies^15^
Reduction in QoL	50% over 7.5 days hospital admission and by 20% for further 30 days recovery	Modelling study.^16^
**Statin therapy costs**		
Standard statin therapy (eg, atorvastatin 20 mg/day, rosuvastatin 5–10 mg/day or simvastatin 40–80 mg/day)	£14.09 to £19.57 per year; £14.35 used in base-case	NHS Drug tariff, December 2021^17^
Higher intensity statin therapy (eg, atorvastatin 40–80 mg/day, rosuvastatin 20–40 mg/day)	£15.91 to £27.91 per year; £21.91 used in base-case	NHS Drug tariff, December 2021^17^
**Statin initiation and monitoring healthcare costs**		
in year of initiation (doctor and nurse consultations; tests of blood lipids, HbA1c, thyroid function)	£54.65	Unit costs for Health and Social Care^18^; NHS reference costs^19^
in subsequent years: a nurse consultation and a blood lipids test (for people with history of CVD)	£12.05	Unit costs for Health and Social Care^18^; NHS reference costs^19^

CVD, cardiovascular disease; HbA1chemoglobin A1C LDLlow-density lipoproteinNHS, National Health Service England; NICE, National Institute for Health and Care Excellence; ORodds ratioQoL, quality of life; RR, relative risk

### Cost-effectiveness of statin therapy

We employed the model to project event risks and survival and summarise life years, quality-adjusted life years (QALYs) and primary and hospital care costs over individuals’ remaining lifetimes (ie, death or 110 years of age) without and with statin therapy and to assess the cost-effectiveness of different statin therapies in categories of older individuals.

#### Base-case analysis

In our base-case analysis, we assessed the cost-effectiveness of lifetime statin therapy from the perspective of the UK National Health Service under a number of key assumptions based on current evidence. First, the reductions in individuals’ LDL-C levels with a particular statin therapy were assumed to correspond to the average proportional reduction achieved with the therapy. Second, we assumed that the relative effects of a particular statin therapy on event risks were independent of duration of therapy or individual person characteristics including age (ie, the overall effects reported in meta-analyses were employed). Third, disease events were assumed not to differ in severity or otherwise, irrespective of statin treatment status. Finally, statin therapy was assumed not to affect the risks of cancer or other non-vascular events,^20^ nor confer any discomfort or disutility beyond the adverse events specified above.

#### Assessment of uncertainty

We ran 500 microsimulations per individual for each set of parameters. We summarised the parameter uncertainty, including uncertainty in effects of statin therapy on vascular and non-vascular events, all event risk equations, QoL and healthcare cost equations in the decision-analytic model using 1000 sets of parameter values, derived using a bootstrap approach, employing sampling with replacement from respective populations.^21^ Values for treatment effects were sampled from lognormal distributions corresponding to the natural logarithm of relative risk reductions with statin therapy.

We report life years and QALYs gained, the additional statin and other healthcare costs (2020/2021 UK£) and the incremental costs per QALY with standard and higher-intensity statin therapies. We discounted future QALYs and costs at 3.5% per year in the summary measures for cost-effectiveness.^22^ We present cost-effectiveness acceptability curves for willingness-to-pay values from £0-£40K/QALY.

#### Sensitivity and scenario analyses

The following parameters were varied. First, in view of the higher uncertainty in the effects of statin therapy in older people, in scenario analyses, we applied relative risk reductions in cardiovascular endpoints per 1 mmol/L LDL-C, informed from data only among: (1) people >75 years old at randomisation and (2) people >75 years old and without prior CVD at randomisation in the individual participant data meta-analysis.^2^ Second, to explore sensitivity to possible double counting of statin effects in the model through its direct effect on vascular death risk and indirect effects through MI and stroke risks, we studied the impact of smaller direct relative risk reduction in cardiovascular death with statin therapy (ie, 7% instead of 12% per 1 mmol/L in LDL-C reduction). Third, to assess sensitivity to variation in major non-vascular disease risk, we ran scenario analyses with a small detrimental or beneficial statin effect on incident cancer, informed by the 95% CI limits reported in a meta-analysis of randomised statin trials.^20^ Fourth, in acknowledgement of substantial rates of statin discontinuation and reinitiation, a scenario analysis assessed statin cost-effectiveness using estimated real-world compliance with statin derived from routine UK data,^23^ with statin effects and costs discontinued with therapy discontinuation. Fifth, to acknowledge the uncertainty concerning any further QoL disutility from taking a daily statin pill, we included analyses with yearly disutility equal to 0.001, 0.002 or 0.005. Sixth, we present scenarios with doubled risk of non-vascular death; with lower general QoL; and both together to assess sensitivity to further reduced potential in older people to benefit from preventive treatment. We also present scenario analyses with only healthcare costs for CVD and incident diabetes included; with higher costs of statin therapy and with 1.5% discount rate for costs and outcomes.

Further details are provided in theonline supplemental methods.

### Patient and public involvement

Three members of the public were involved in the study management and steering groups. Study methods and results were also discussed in separate sessions with our lay members who helped us refine the study methodology and approach to presenting study findings.

## Results

The baseline characteristics of participants ≥70 years old in the UK Biobank and Whitehall II studies in categories by prior CVD are presented in table 2 and online supplemental table 2. There were 15 019 (52% men; mean age 72.5 years) participants without CVD and 5103 (66% men; mean age 72.9 years) with history of CVD. Among participants without and with prior CVD, 29% and 58%, respectively, were prescribed a statin at baseline and the derived untreated mean LDL-C levels were 4.2 mmol/L (SD 0.78 mmol/L) and 4.3mmol/L (SD 0.98 mmol/L), respectively.

**Table 2:** Baseline characteristics of UK Biobank and Whitehall II participants 70 years and older

	Participants without CVD	Participants with prior CVD
**Number of participants**	15 019	5103
Age, years	72.5 (2.5)	72.9 (2.7)
≥75 years	3149 (21%)	1345 (26%)
Male sex	7838 (52%)	3389 (66%)
Ethnicity		
White	14 686 (98%)	4916 (96%)
Black	55 (0%)	13 (0%)
South Asian	166 (1%)	134 (3%)
Other*	112 (1%)	40 (1%)
Townsend socioeconomic deprivation		
Quintile 1 (least deprived)	6370 (42%)	1926 (38%)
Quintile 2	3066 (20%)	1005 (20%)
Quintile 3	2879 (19%)	1123 (22%)
Quintile 4	1774 (12%)	693 (14%)
Quintile 5	930 (6%)	356 (7%)
Smoking status		
Never	8523 (57%)	2486 (49%)
Former smoker	6034 (40%)	2444 (48%)
Current smoker	462 (3%)	173 (3%)
Physical activity		
High	5257 (35%)	1694 (33%)
Moderate	5486 (37%)	1934 (38%)
Low	1806 (12%)	688 (13%)
Missing	2470 (16%)	787 (15%)
Unhealthy diet (incl. uncertain)	4363 (29%)	1765 (35%)
BMI (kg/m†)	27 (4.1)	27 (4.3)
<18.5	99 (1%)	24 (0%)
18.5–25	5642 (38%)	1478 (29%)
25–30	6674 (44%)	2380 (47%)
30–35	2084 (14%)	941 (18%)
35–40	422 (3%)	222 (4%)
40+	98 (1%)	58 (1%)
LDL-C (mmol/L)	3.7 (0.65)	3.2 (0.74)
HDL-C (mmol/L)	1.7 (0.31)	1.6 (0.32)
On statin treatment	4289 (29%)	2979 (58%)
Derived untreated LDL-C (mmol/L)†	4.2 (0.78)	4.3 (0.98)
Creatinine (umol/L)	78 (13)	84 (19)
Systolic BP (mm Hg)	146 (18)	142 (19)
Diastolic BP (mm Hg)	79 (10)	77 (11)
Treated hypertension	4076 (27%)	2631 (52%)
Prior diabetes	1154 (8%)	782 (15%)
Prior cancer	2040 (14%)	774 (15%)
Severe mental illness	1206 (8%)	452 (9%)
Prior CVD history		
MI only		103 (2%)
PAD only		380 (7%)
Other CHD‡ only		2910 (57%)
Stroke only		343 (7%)
Two or more of MI, PAD other CHD or stroke		1367 (27%)

Values are mean (SD) or number (%).

* Other ethnicity includes Chinese, Mixed, White and Black Caribbean, White and Black African, White and Asian, any other mixed background and other ethnic group.

† Adjusted for use of statin treatment at baseline by statin type and dose.

‡ Other CHD includes acute rheumatic fever, chronic rheumatic heart diseases, hypertensive heart disease, angina pectoris, other acute ischaemic heart disease, chronic ischaemic heart disease, pulmonary heart disease and other form of heart disease.

BPblood pressureCHD, coronary heart disease; CVD, cardiovascular disease; HDL, high-density lipoprotein; LDL, low-density lipoprotein; MImyocardial infarctionPAD, peripheral arterial disease

**Figure 1:**
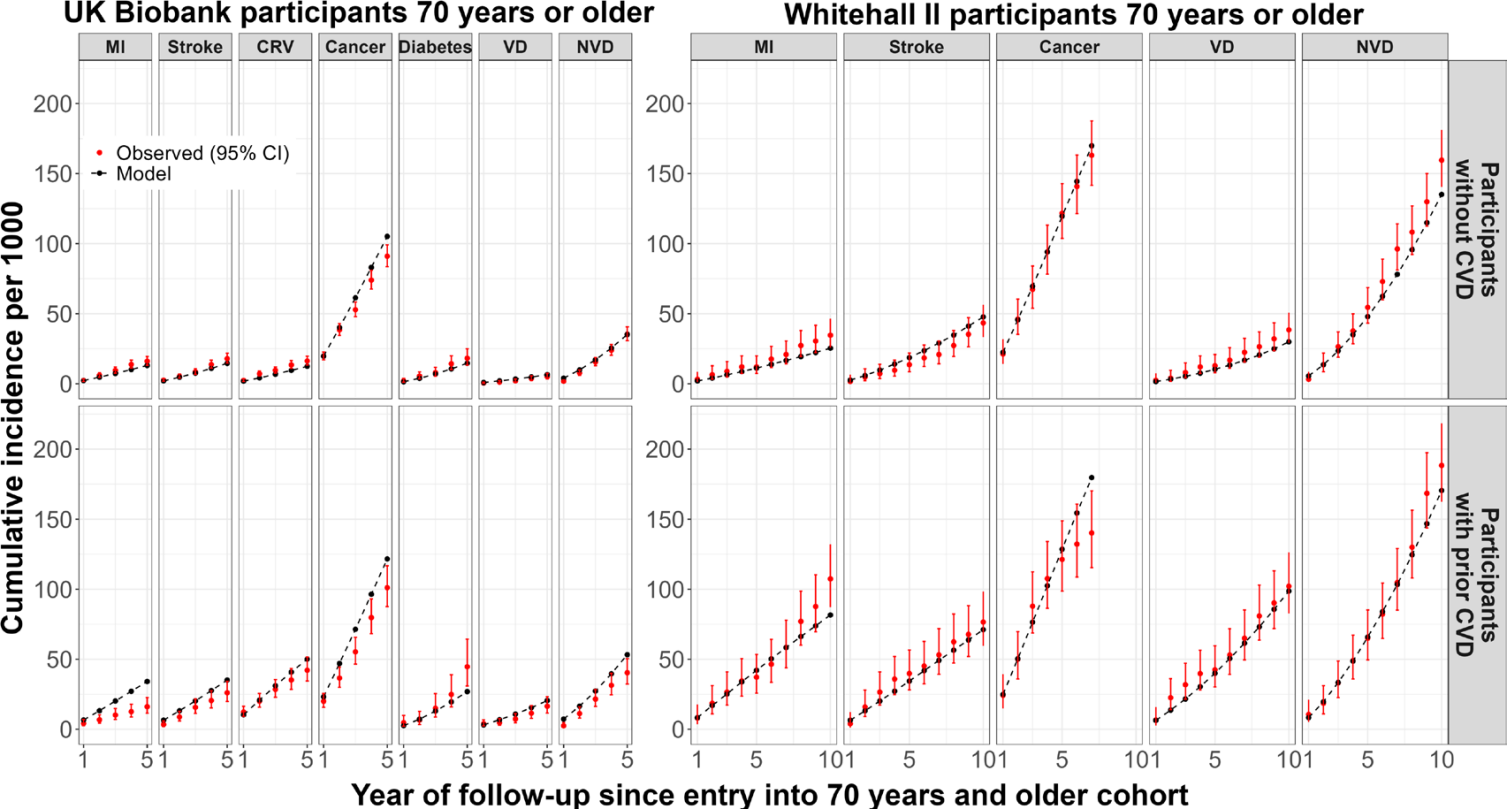
CVD microsimulation model validation among UK Biobank and Whitehall II participants 70 years and older. In the Whitehall II study, no linked data for CRV and diabetes were available and, therefore, no model validation performed for CRV and diabetes. CRV, coronary revascularisation; CVD, cardiovascular disease; MI, myocardial infarction; NVD, nonvascular death; VD, vascular disease.

In model validation, the cumulative event rates predicted by the CVD microsimulation model, using the baseline characteristics of participants ≥70 years old, corresponded mostly well to the observed rates of cardiovascular and non-vascular events in categories of participants by prior CVD, respectively, though higher MI risks, but not cardiovascular death risks, were predicted among participants with prior CVD in UK Biobank but not in Whitehall II study (figure 1).

**Figure 2:**
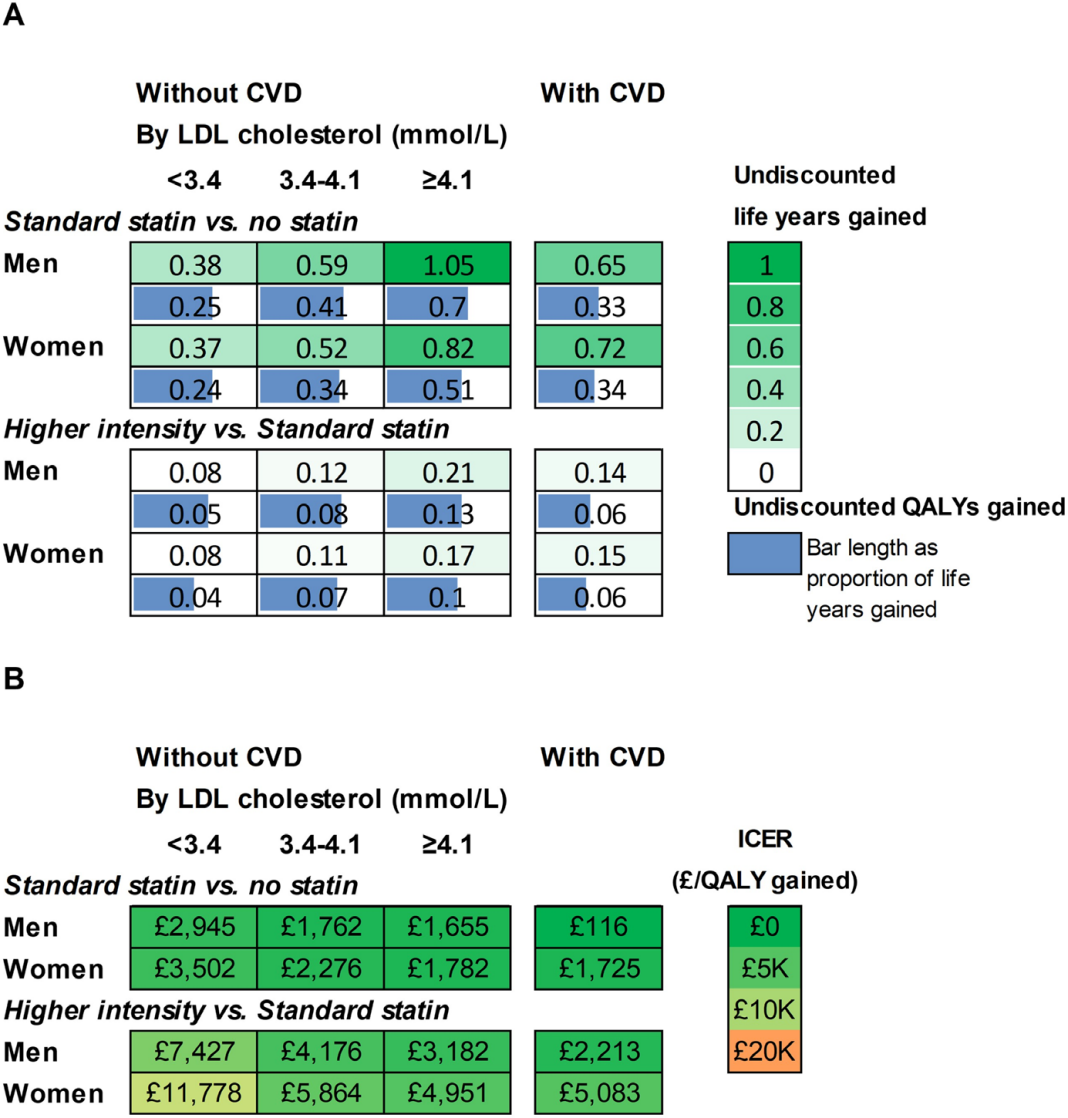
Life years and QALYs gained (**A**) and cost-effectiveness (**B**) of lifetime statin therapy in categories by prior cardiovascular disease, sex and pre-treatment LDL cholesterol level. Incremental Cost-Effectiveness Ratio (ICER) is the ratio of the incremental costs divided by the incremental QALYs with costs and QALYs discounted at 3.5% per year. CVD, cardiovascular disease; LDL, low density lipoprotein; QALY, quality-adjusted life years.

**Figure 3:**
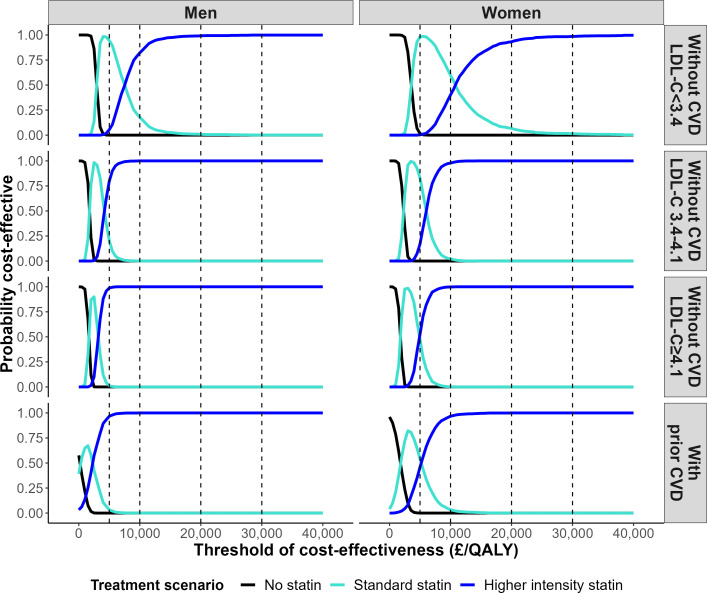
Probability that lifetime statin therapy is cost-effective in categories by prior cardiovascular disease, sex and pre-treatment LDL cholesterol level. The probability that the treatment scenario provides the highest QALYs gain at the particular threshold of cost-effectiveness plotted. CVD, cardiovascular disease; LDL-C, low-density lipoprotein cholesterol; QALY, quality-adjusted life years.

In participant categories by sex, prior CVD and LDL-C level, standard statin therapy was projected to increase individual survival (undiscounted) by 0.37 to 1.05 life years (0.24 to 0.7 QALYs), and higher intensity statin therapy by a further 0.08 to 0.21 life years (0.04 to 0.13 QALYs) (figure 2A, online supplemental tables 3 and 4). Across these categories, the incremental cost per QALY gained for standard statin therapy compared with no statin ranged from £116 to £3502 and that for higher intensity compared with standard statin from £2213 to £11 778 per QALY (figure 2B). The analyses of parameter uncertainty indicated that at £20 000/QALY willingness to pay threshold, higher intensity statin therapy had a very high probability of being cost-effective across all categories of men and women ≥70 years old (figure 3). The probability that statin therapy was cost-effective for people ≥70 years old remained high even at a cost-effectiveness threshold of £5K/QALY. However, at this lower threshold, the standard statin therapy had the highest probability of being cost-effective among women with a pretreatment LDL-C lower than 4.1 mmol/L and among men with a pretreatment LDL-C lower than 3.4 mmol/L (figure 3).

**Figure 4:**
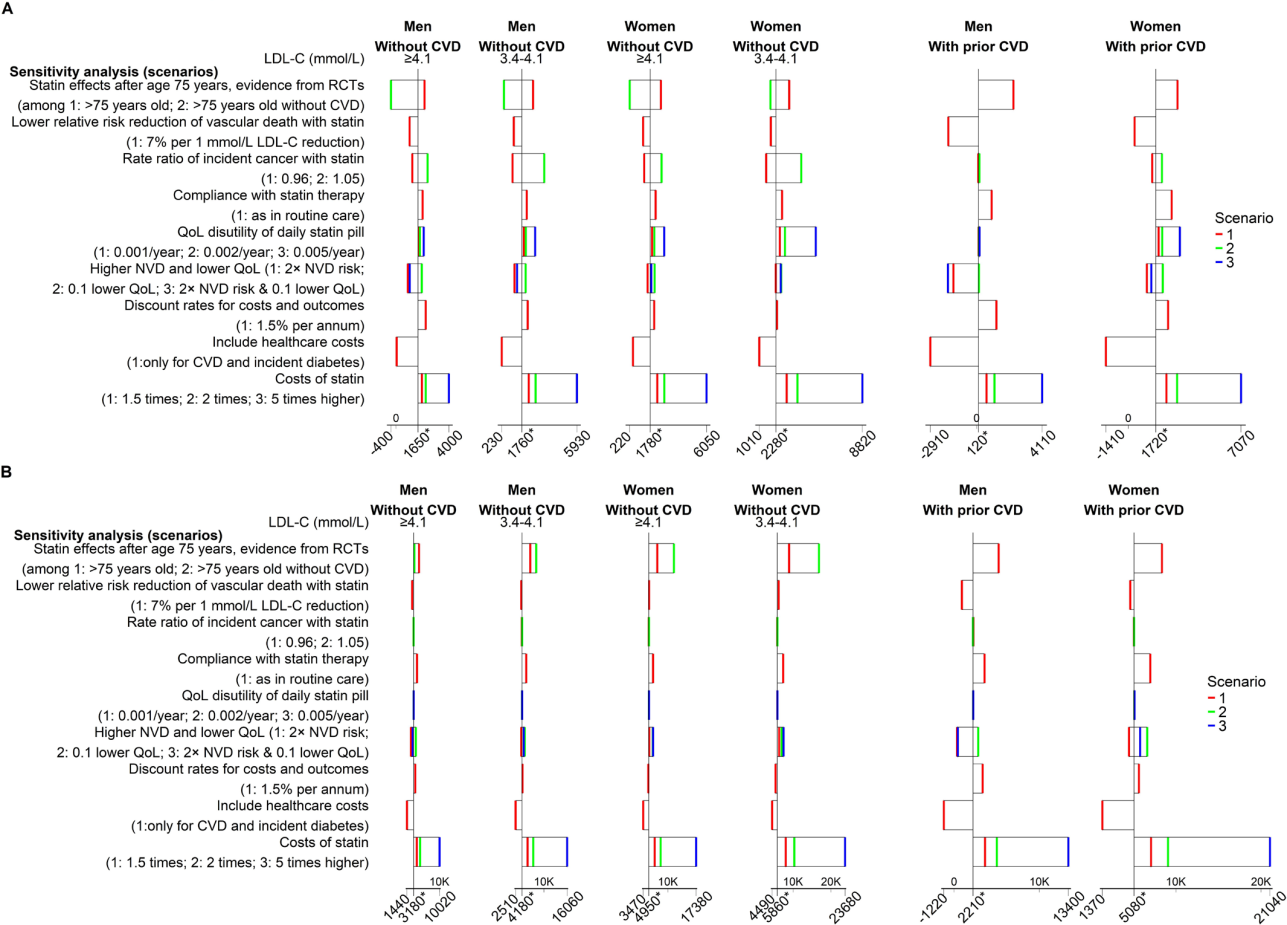
Sensitivity analyses of cost-effectiveness of statin therapy for people 70 years or older. (A) Incremental cost (£) per QALY gained (standard statin vs no statin). (B) Incremental cost (£) per QALY gained (higher intensity vs standard statin). See online supplemental methods table 7 for description of sensitivity analyses. The * on the horizontal axes represent the base-case analysis. CVD, cardiovascular disease; LDL, low-density lipoprotein; NVD, nonvascular death; QALY, quality-adjusted life year; QoL, quality of life.

These cost-effectiveness results remained robust in a wide range of sensitivity analyses (figure 4, online supplemental table 5) with higher sensitivity noted for a higher intensity statin at a five times higher price. In particular, although reduced gains in QALYs were projected, standard statin therapy remained cost-effective in people ≥70 years old if relative risk reductions after age 75 were equal to those reported in the subgroup of participants >75 years old, or indeed in the subgroup of participants >75 years old without CVD at randomisation, in the CTTC meta-analysis (figures4  5 and online supplemental figure 1). Higher intensity statin therapy remained cost-effective among older people with pretreatment cholesterol levels 3.4 mmol/L or higher. In these scenario analyses with lower CVD risk reductions with statin therapy, the probability of standard or higher intensity statin therapy being cost-effective remained higher than no statin therapy in all categories of older people but was substantially reduced among older women with lower LDL-C levels.

**Figure 5:**
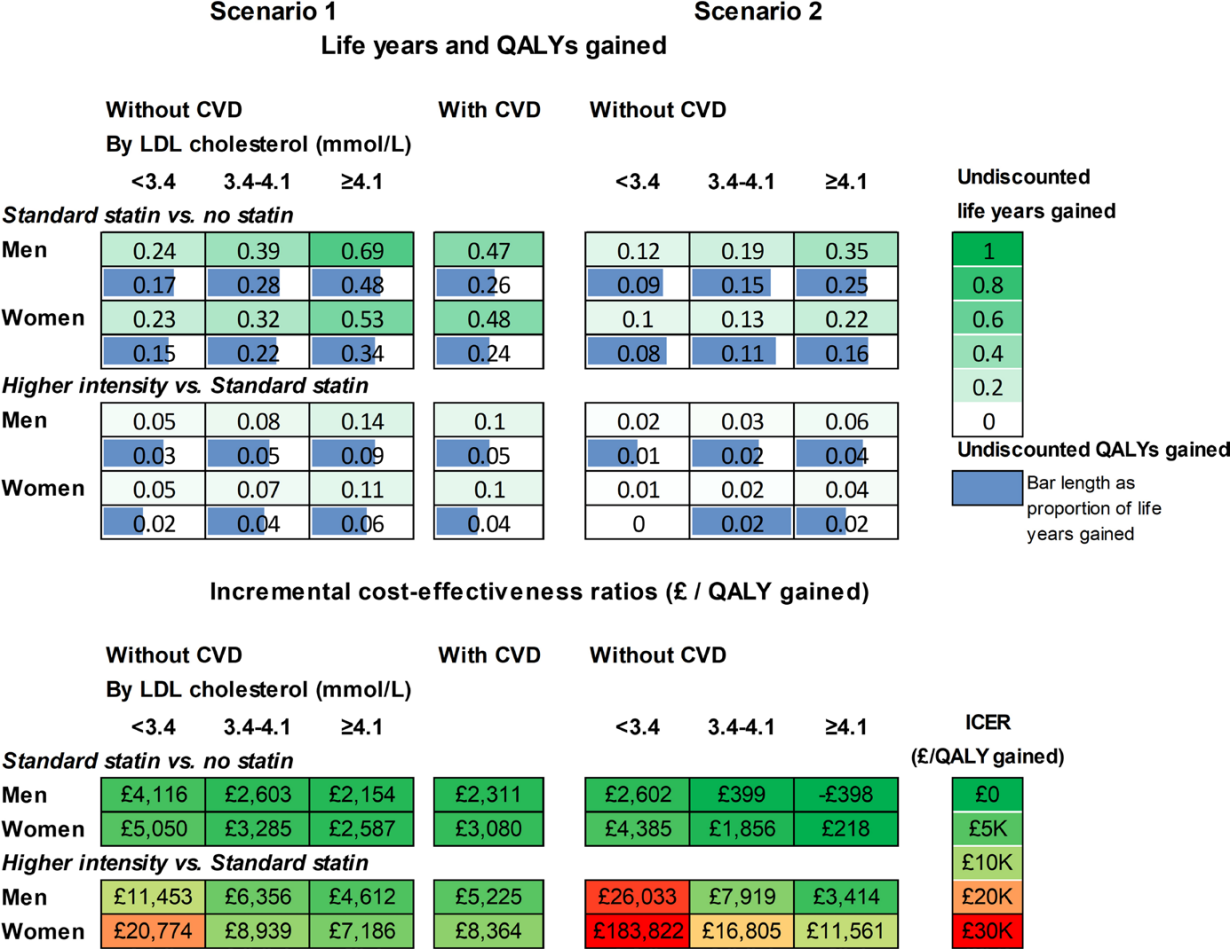
Life years and QALYs gained and cost-effectiveness of lifetime statin therapy in older people: scenario analyses with CVD reductions with statin therapy in people>75 years old informed from effects of statin therapy among participants>75 years old (Scenario 1) or >75 years old without CVD (Scenario 2) from Cholesterol Treatment Trialists’ collaborative meta-analysis. Statin effects up to age 75 as in base-case analysis; statin effect thereafter as per respective scenario analysis. CVD, cardiovascular disease; ICER, Incremental Cost-Effectiveness Ratio with costs and QALYs discounted at 3.5% per year; LDL, low-density lipoprotein; QALY, quality-adjusted life year.

## Discussion

This assessment of the lifetime effects and cost-effectiveness of statin therapy in people ≥70 years old in the UK used contemporary patient data, a validated CVD microsimulation model and a meta-analysis of the effects of statin treatment across age categories. It concluded that lifetime statin treatment increased quality-of-life-adjusted survival in older men and women and, at UK cost of generic statins, was highly cost-effective for all, irrespective of their CVD history or LDL-C level. Higher intensity statin therapy was the strategy likely to bring the highest health benefits cost-effectively, although standard statin regimens would achieve most of these benefits. These findings remained robust in sensitivity analyses with smaller cardiovascular risk reductions with statin therapy, though smaller benefits were projected and standard statin therapy became the preferred option for older people with LDL-C levels <3.4 mmol/L.

In this analysis, we used the overall relative risk reductions in cardiovascular events per 1 mmol/L LDL-C reduction with statin therapy given the similar relative risk reductions across age categories in the individual participant meta-analysis of statin trials.^2^ The meta-analysis, however, noted trends towards smaller proportional reductions in major coronary events and vascular deaths in older people. Data were particularly limited among participants >75 years old without prior CVD, where there was no direct evidence for statistically significant cardiovascular risk reductions with statin therapy. In the present report, two scenario analyses assessed the sensitivity of findings to the size of statin effects using relative risk reductions in cardiovascular events in the meta-analysis (1) among participants >75 years old, and (2) among participants >75 years old without prior CVD at randomisation.^2^ In both scenarios, despite smaller net health benefits, statin therapy remained cost-effective although with larger uncertainty.

We previously reported that statin therapy, at generic prices, is highly cost-effective in UK across patients 40–70 years old irrespective of their sex, age, CVD risk and LDL-C level.^21^ Here, we extend this work to older people and indicate that, although the gains in QALYs are smaller, the additional costs are also lower, and the incremental cost per QALY remains highly attractive. Moreover, with a substantially higher CVD risk (99% of ≥70 years old UK Biobank participants without prior CVD had estimated 10 year CVD risk ≥10%; and 88% had 10-year CVD risk ≥15%, data not shown), the level of risk is irrelevant in guiding statin treatment decisions in older people.

This reassessment of statins’ value in the contemporary older UK population confirms findings of earlier cost-effectiveness studies^8 9^ and reaffirms that, despite substantial reductions in CVD incidence and mortality over the last decades, statins remain a cornerstone in CVD prevention in this population. Our findings differ from an earlier study of cost-effectiveness of statin therapy for the primary prevention of CVD in people ≥75 years old, which reported that, although statin treatment was highly cost-effective, even a small hypothetical increase in a geriatric-specific adverse effect (ie, reducing disability-adjusted life years by 0.003–0.004) would offset its cardiovascular benefit.^7^ In our study, the known small excesses of myopathy, rhabdomyolysis and incident diabetes with statin treatment were explicitly integrated, and our findings remained robust to hypothetical further statin-associated reductions in QoL up to 0.005 QALY/ year and to lower statin efficacy, suggesting that the value of statin therapy for older people is more certain than implied. It is important to also underline that high-quality randomised evidence indicate that the vast majority of adverse effects reported on statin therapy were also reported in the absence of statin therapy,^24 25^ indicating serious misattribution of adverse effects in observational and uncontrolled studies.

Our results indicate that older people are likely to cost-effectively benefit from statin treatment. Statin treatment rates in our ≥70 years old cohort (29% among people without CVD to 58% among people with prior CVD) were similar to statin treatment rates reported by the Health Survey for England.^26^ Hence, from the 9.1 million adults ≥70 years old in UK,^27^ a third of them with prior CVD,^26^ just over 40%, or less than 4 million, are receiving statin treatment. While further evidence for statins effects in older people will be helpful, the robustness of the findings to variations in key parameters suggests that delaying statin treatment in the millions of older people while awaiting new evidence is unjustifiable.

Our study has a number of strengths. We used a contemporary UK CVD model, developed using a large and rich population biobank with demonstrable ability to predict cardiovascular and mortality risks in older people. We used the baseline characteristics of more than 20 000 people ≥70 years old to evaluate lifetime benefits and cost-effectiveness of statin therapy. A further strength of our analysis is the use of synthesised randomised evidence for the effects of statin therapy by age that allowed us to study the robustness of our findings to somewhat smaller reductions in cardiovascular risks in older people. Finally, the reported excesses in myopathy, rhabdomyolysis and incident diabetes with standard and higher intensity statin therapy were integrated allowing the net effects of treatment to be fully assessed.

The study has some limitations. First, the majority of our data is among people aged 70 to early 80s. Our findings, however, were very similar in participants 70–75 and ≥75 years old (results not shown), which suggest that they are generalisable to much older people. Second, our model and results are based on population cohorts, in which the healthy volunteer effect may limit generalisability. To address this limitation, the model used a broad range of socioeconomic, lifestyle and clinical characteristics that allow generalisations to populations with different distributions of these characteristics. Moreover, statin therapy remained cost-effective in scenario analyses with substantially higher risk of non-vascular death and lower QoL. Third, a small excess in milder muscle symptoms was recently reported with statin treatment across randomised studies with excess confined to the first year of treatment.^28^ The sensitivity analyses suggest that this adverse effect is unlikely to materially alter statin’s cost-effectiveness. Fourth, two ongoing large statin trials, scheduled to complete in 2026, will add valuable further data to the direct evidence of effects of statin therapy in people aged ≥75 years without atherosclerotic CVD.^29 30^ Fifth, missing baseline data were imputed using a single imputation. Moreover, while the model performance was good for most participant categories, endpoints and across the two datasets, there were some deviations. Therefore, it is possible that the uncertainty may be larger than reported by the model. However, the consistency of cost-effectiveness results across categories of participants and across a broad range of sensitivity analyses for key parameters indicate that our general findings are robust.

In conclusion, this study reports that statin therapy is highly likely to be cost-effective in older people, although there was greater uncertainty among older people without CVD in scenario analysis with substantially smaller CVD risk reductions with statin therapy. While further randomised evidence will be helpful, the robustness of these findings indicates that older people are likely to benefit cost-effectively from statin therapy and should be considered for treatment.

## supplementary material

10.1136/heartjnl-2024-324052 online supplemental file 1

## Data Availability

Data may be obtained from a third party and are not publicly available.
