# Supplementary material for: Lifetime effects and cost-effectiveness of statin therapy for older people in the United Kingdom: a modelling study
Source: Heart. 2024 Sep 10;110(21):e324052. doi: 10.1136/heartjnl-2024-324052 (PMC11503164; doi:10.1136/heartjnl-2024-324052)
Supplement: online supplemental file 1 [file heartjnl-110-21-s001.docx]

**Lifetime effects and cost-effectiveness of statin therapy for older people in the United Kingdom: a modelling study**

**SUPPLEMENTAL MATERIAL**

Table of Contents

[Supplemental Table 1: Proportional reductions in LDL cholesterol with statin regimens 2](#_Toc173448846)

[Supplemental Table 2: Baseline characteristics of UK Biobank and Whitehall II participants 70 years and older 3](#_Toc173448847)

[Supplemental Table 3: Predicted incremental health outcomes, costs and cost-effectiveness of standard statin treatment compared to no statin, in categories by sex, prior cardiovascular disease and pre-treatment LDL cholesterol level 5](#_Toc173448848)

[Supplemental Table 4: Predicted incremental health outcomes, costs and cost-effectiveness of higher intensity statin treatment compared to standard statin, in categories by sex, prior cardiovascular disease and pre-treatment LDL cholesterol level 7](#_Toc173448849)

[Supplemental Table 5: Predicted discounted incremental health outcomes, costs and cost-effectiveness of standard statin treatment compared to no statin, in categories by sex, prior cardiovascular disease and pre-treatment LDL cholesterol level: base case and selected sensitivity analyses 9](#_Toc173448850)

[Supplemental Figure 1: Probability of statin therapy being cost-effective in older people in scenario analyses with CVD reductions with statin therapy in people>75 years old informed from effects of statin therapy among participants >75 years old (Scenario 1) or >75 years old without CVD (Scenario 2) from Cholesterol Treatment Trialists’ collaborative meta-analysis 11](#_Toc173448851)

[Supplemental methods 12](#_Toc173448852)

[The CVD micro-simulation model 12](#_Toc173448853)

[Identifying participants 70 years and older 12](#_Toc173448854)

[Handling missing data 15](#_Toc173448855)

[Derivation of pre-treatment LDL cholesterol levels for statin-treated UK Biobank and WHITEHALL II participants 18](#_Toc173448856)

[Integrating treatment effects of statin therapy in the CVD model 18](#_Toc173448857)

[Specification of sensitivity and scenario analyses 19](#_Toc173448858)

[Supplemental material references 22](#_Toc173448859)

## Supplemental Table 1: Proportional reductions in LDL cholesterol with statin regimens

|  | **% reduction in LDL cholesterol^1^** | | | | |
| --- | --- | --- | --- | --- | --- |
| **Dose (mg/day)** | **5mg** | **10mg** | **20mg** | **40mg** | **80mg** |
| Fluvastatin | 10% | 15% | 21% | 27% | 33% |
| Pravastatin | 15% | 20% | 24% | 29% | 33% |
| Simvastatin | 23% | 27% | 32% | 37% | 42% |
| Atorvastatin | 31% | 37% | 43% | 49% | 55% |
| Rosuvastatin | 38% | 43% | 48% | 53% | 58% |

^1^Based on Law et al. (1)

## Supplemental Table 2: Baseline characteristics of UK Biobank and Whitehall II participants 70 years and older

|  | **UK Biobank** | | **Whitehall II** | |
| --- | --- | --- | --- | --- |
|  | **Participants without CVD** | **Participants with prior CVD** | **Participants without CVD** | **Participants with prior CVD** |
| **Number of participants** | **13,772** | **4,349** | **1,247** | **754** |
| Age, years | 72.4 (2.6) | 72.7 (2.7) | 73.6 (2.2) | 73.8 (2.2) |
| Male sex | 7009 (51%) | 2874 (66%) | 829 (66%) | 515 (68%) |
| Ethnicity |  |  |  |  |
| White | 13532 (98%) | 4259 (98%) | 1154 (93%) | 657 (87%) |
| Black | 55 (0%) | 13 (0%) |  |  |
| South Asian | 73 (1%) | 37 (1%) | 93 (7%)^2^ | 97 (13%)^2^ |
| Others^1^ | 112 (1%) | 40 (1%) |  |  |
| Townsend socioeconomic deprivation |  |  |  |  |
| Quintile 1 (least deprived) | 6370 (46%) | 1926 (44%) | 0 | 0 |
| Quintile 2 | 2958 (21%) | 959 (22%) | 108 (9%) | 46 (6%) |
| Quintile 3 | 2032 (15%) | 612 (14%) | 847 (68%) | 511 (68%) |
| Quintile 4 | 1483 (11%) | 497 (11%) | 291 (23%) | 196 (26%) |
| Quintile 5 | 929 (7%) | 355 (8%) | 1 (0%) | 1 (0%) |
| Smoking status |  |  |  |  |
| Never | 7870 (57%) | 2112 (49%) | 653 (52%) | 374 (50%) |
| Former smoker | 5508 (40%) | 2110 (49%) | 526 (42%) | 334 (44%) |
| Current smoker | 394 (3%) | 127 (3%) | 68 (5%) | 46 (6%) |
| Physical activity |  |  |  |  |
| High | 4794 (35%) | 1452 (33%) | 463 (37%) | 242 (32%) |
| Moderate | 4779 (35%) | 1501 (35%) | 707 (57%) | 433 (57%) |
| Low | 1762 (13%) | 641 (15%) | 44 (4%) | 47 (6%) |
| Missing | 2437 (18%) | 755 (17%) | 33 (3%) | 32 (4%) |
| Unhealthy diet (incl. uncertain) | 3566 (26%) | 1287 (30%) | 797 (64%) | 478 (63%) |
| BMI (kg/m^2^) | 27 (4) | 27 (4.2) | 27 (4.3) | 28 (4.5) |
| <18.5 | 86 (1%) | 20 (0%) | 13 (1%) | 4 (1%) |
| 18.5-25 | 5187 (38%) | 1263 (29%) | 455 (36%) | 215 (29%) |
| 25-30 | 6138 (45%) | 2054 (47%) | 536 (43%) | 326 (43%) |
| 30-35 | 1892 (14%) | 785 (18%) | 192 (15%) | 156 (21%) |
| 35-40 | 384 (3%) | 180 (4%) | 38 (3%) | 42 (6%) |
| 40+ | 85 (1%) | 47 (1%) | 13 (1%) | 11 (1%) |
| LDL-C (mmol/L) | 3.7 (0.59) | 3.3 (0.65) | 3 (0.93) | 2.6 (0.9) |
| HDL-C (mmol/L) | 1.7 (0.3) | 1.6 (0.3) | 1.7 (0.42) | 1.6 (0.43) |
| On statin treatment | 3870 (28%) | 2498 (57%) | 419 (34%) | 481 (64%) |
| Derived pre-treated LDL (mmol/L)^3^ | 4.2 (0.72) | 4.4 (0.9) | 3.4 (0.99) | 3.7 (1.2) |
| Creatinine (umol/L) | 77 (12) | 81 (13) | 88 (24) | 96 (38) |
| Systolic BP (mmHg) | 147 (18) | 145 (18) | 129 (16) | 128 (17) |
| Diastolic BP (mmHg) | 80 (10) | 78 (10) | 71 (10) | 69 (10) |
| Treated hypertension | 3593 (26%) | 2098 (48%) | 483 (39%) | 533 (71%) |
| Prior diabetes | 939 (7%) | 578 (13%) | 215 (17%) | 204 (27%) |
| Prior cancer | 1877 (14%) | 680 (16%) | 163 (13%) | 94 (12%) |
| Severe mental illness | 1192 (9%) | 443 (10%) | 14 (1%) | 9 (1%) |
| Prior CVD history |  |  |  |  |
| MI only |  | 69 (2%) |  | 34 (5%) |
| PAD only |  | 354 (8%) |  | 26 (3%) |
| Stroke only |  | 315 (7%) |  | 28 (4%) |
| Other CHD^4^ only |  | 2454 (56%) |  | 456 (60%) |
| Two or more of MI, PAD, other CHD or stroke |  | 1157 (27%) |  | 210 (28%) |

Values are mean (SD) or number (%). CHD, coronary heart disease; CVD, cardiovascular disease; HDL, high density lipoprotein; LDL, low density lipoprotein; PAD, peripheral arterial disease; SD, standard deviation.

^1^Other ethnicity includes Chinese, Mixed, White and Black Caribbean, White and Black African, White and Asian, Any other mixed background and other ethnic group.

^2^Whitehall II only has two categories of ethnicity, White and non-White. We made the assumption that all non-White were South Asians, the second largest ethnicity group in the UK.

^3^Adjusted for use of statin treatment at baseline by statin type and dose.

^4^Other CHD includes acute rheumatic fever, chronic rheumatic heart diseases, hypertensive heart disease, angina pectoris, other acute ischaemic heart disease, chronic ischaemic heart disease, pulmonary heart disease and other form of heart disease.

## Supplemental Table 3: Predicted incremental health outcomes, costs and cost-effectiveness of standard statin treatment compared to no statin, in categories by sex, prior cardiovascular disease and pre-treatment LDL cholesterol level

| **Sex** | **Men** | | | | **Women** | | | |
| --- | --- | --- | --- | --- | --- | --- | --- | --- |
| **CVD history and LDL-C levels** | **Without CVD, LDL-C < 3.4** | **Without CVD, LDL-C 3.4-4.1** | **Without CVD, LDL-C ≥4.1** | **With prior CVD** | **Without CVD, LDL-C < 3.4** | **Without CVD, LDL-C 3.4-4.1** | **Without CVD, LDL-C ≥4.1** | **With prior CVD** |
| **Incremental Life years (95% CI)** | 0.38 (0.3, 0.46) | 0.59 (0.48, 0.71) | 1.05 (0.87, 1.22) | 0.65 (0.52, 0.81) | 0.37 (0.29, 0.46) | 0.52 (0.4, 0.64) | 0.82 (0.65, 0.97) | 0.72 (0.56, 0.9) |
| **Incremental Life years, discounted (95% CI)** | 0.18 (0.15, 0.22) | 0.28 (0.23, 0.33) | 0.51 (0.43, 0.59) | 0.35 (0.28, 0.43) | 0.15 (0.12, 0.19) | 0.21 (0.17, 0.26) | 0.34 (0.27, 0.41) | 0.33 (0.26, 0.41) |
| **Incremental QALYs (95% CI)** | 0.25 (0.2, 0.31) | 0.41 (0.33, 0.49) | 0.7 (0.58, 0.82) | 0.33 (0.25, 0.42) | 0.24 (0.18, 0.29) | 0.34 (0.26, 0.42) | 0.51 (0.41, 0.61) | 0.34 (0.26, 0.44) |
| **Incremental QALYs, discounted (95% CI)** | 0.12 (0.1, 0.15) | 0.2 (0.16, 0.24) | 0.35 (0.29, 0.4) | 0.18 (0.14, 0.23) | 0.1 (0.08, 0.12) | 0.14 (0.11, 0.18) | 0.22 (0.18, 0.27) | 0.16 (0.12, 0.21) |
| **Incremental Hospital cost (95% CI)** | 280 (91, 466) | 371 (99, 636) | 885 (458, 1284) | -321 (-787, 187) | 123 (-63, 322) | 123 (-108, 376) | 262 (-76, 600) | -172 (-685, 385) |
| **Incremental Hospital cost, Discounted (95% CI)** | -3 (-88, 84) | -37 (-158, 86) | 54 (-144, 243) | -583 (-840, -305) | -35 (-111, 52) | -63 (-157, 41) | -79 (-213, 70) | -392 (-639, -122) |
| **Incremental Primary care cost (95% CI)** | 250 (181, 318) | 298 (212, 384) | 610 (466, 747) | 485 (320, 675) | 255 (164, 340) | 265 (172, 362) | 475 (327, 609) | 567 (363, 811) |
| **Incremental Primary care cost, discounted (95% CI)** | 103 (71, 136) | 112 (73, 152) | 248 (180, 311) | 225 (138, 326) | 95 (58, 131) | 92 (54, 132) | 172 (115, 228) | 222 (130, 331) |
| **Statin cost (95% CI)** | 285 (276, 293) | 297 (288, 307) | 292 (282, 301) | 248 (237, 260) | 355 (345, 363) | 365 (355, 373) | 365 (354, 373) | 329 (314, 344) |
| **Statin cost, discounted (95% CI)** | 199 (195, 203) | 205 (201, 210) | 203 (199, 207) | 180 (174, 185) | 231 (227, 234) | 236 (232, 239) | 236 (232, 239) | 219 (212, 225) |
| **Statin Monitoring cost (95% CI)** | 74 (72, 76) | 74 (72, 77) | 79 (76, 82) | 258 (249, 268) | 73 (71, 76) | 72 (69, 74) | 74 (71, 77) | 328 (315, 342) |
| **Statin Monitoring cost, discounted (95% CI)** | 66 (65, 67) | 66 (65, 67) | 69 (68, 70) | 199 (195, 204) | 65 (64, 66) | 64 (63, 65) | 65 (64, 66) | 233 (228, 239) |
| **Incremental Total cost (£) (95% CI)** | 889 (638, 1134) | 1040 (689, 1386) | 1866 (1286, 2392) | 670 (71, 1355) | 806 (539, 1074) | 825 (505, 1170) | 1175 (696, 1642) | 1052 (347, 1834) |
| **Incremental Total cost, discounted (£) (95% CI)** | 365 (251, 480) | 347 (190, 505) | 574 (313, 821) | 21 (-300, 381) | 355 (246, 472) | 328 (200, 470) | 394 (201, 597) | 282 (-37, 632) |
| **ICER (£/QALY) (95% CI)** | 2945 (2173, 3766) | 1762 (1060, 2390) | 1655 (1020, 2185) | 116 (-1977, 1839) | 3502 (2513, 4783) | 2276 (1502, 3095) | 1782 (1054, 2490) | 1725 (-275, 3531) |

CI, confidence interval. CVD, cardiovascular disease. ICER, Incremental Cost-Effectiveness Ratio with costs and QALYs discounted at 3.5% per year. LDL, low density lipoprotein. QALY, quality-adjusted life years.

## Supplemental Table 4: Predicted incremental health outcomes, costs and cost-effectiveness of higher intensity statin treatment compared to standard statin, in categories by sex, prior cardiovascular disease and pre-treatment LDL cholesterol level

| **Sex** | **Men** | | | | **Women** | | | |
| --- | --- | --- | --- | --- | --- | --- | --- | --- |
| **CVD history and LDL-C levels** | **Without CVD, LDL-C < 3.4** | **Without CVD, LDL-C 3.4-4.1** | **Without CVD, LDL-C ≥4.1** | **With prior CVD** | **Without CVD, LDL-C < 3.4** | **Without CVD, LDL-C 3.4-4.1** | **Without CVD, LDL-C ≥4.1** | **With prior CVD** |
| **Incremental Life years (95% CI)** | 0.08 (0.05, 0.1) | 0.12 (0.09, 0.15) | 0.21 (0.17, 0.25) | 0.14 (0.1, 0.18) | 0.08 (0.06, 0.1) | 0.11 (0.08, 0.14) | 0.17 (0.13, 0.21) | 0.15 (0.11, 0.2) |
| **Incremental Life years, discounted (95% CI)** | 0.04 (0.02, 0.05) | 0.06 (0.04, 0.07) | 0.1 (0.08, 0.12) | 0.07 (0.05, 0.09) | 0.03 (0.02, 0.04) | 0.05 (0.03, 0.06) | 0.07 (0.05, 0.08) | 0.07 (0.05, 0.09) |
| **Incremental QALYs (95% CI)** | 0.05 (0.03, 0.06) | 0.08 (0.06, 0.1) | 0.13 (0.1, 0.16) | 0.06 (0.04, 0.09) | 0.04 (0.02, 0.06) | 0.07 (0.05, 0.09) | 0.1 (0.07, 0.12) | 0.06 (0.04, 0.09) |
| **Incremental QALYs, discounted (95% CI)** | 0.02 (0.01, 0.03) | 0.04 (0.03, 0.05) | 0.06 (0.05, 0.08) | 0.03 (0.02, 0.05) | 0.02 (0.01, 0.03) | 0.03 (0.02, 0.04) | 0.04 (0.03, 0.05) | 0.03 (0.02, 0.04) |
| **Incremental Hospital cost (95% CI)** | 79 (26, 127) | 89 (25, 164) | 187 (86, 295) | -31 (-143, 94) | 62 (11, 124) | 48 (-6, 114) | 93 (10, 177) | 18 (-114, 164) |
| **Incremental Hospital cost, Discounted (95% CI)** | 9 (-14, 34) | -1 (-29, 34) | 14 (-30, 65) | -99 (-158, -33) | 10 (-13, 39) | -2 (-25, 25) | 5 (-30, 41) | -51 (-112, 17) |
| **Incremental Primary care cost (95% CI)** | 99 (64, 141) | 105 (71, 146) | 184 (130, 241) | 156 (105, 213) | 120 (70, 187) | 105 (65, 155) | 172 (113, 243) | 189 (120, 268) |
| **Incremental Primary care cost, discounted (95% CI)** | 47 (28, 71) | 46 (27, 68) | 81 (54, 113) | 78 (50, 109) | 53 (28, 86) | 43 (24, 66) | 71 (42, 105) | 82 (50, 119) |
| **Statin cost (95% CI)** | 152 (147, 157) | 159 (154, 165) | 159 (153, 164) | 134 (128, 140) | 189 (184, 193) | 195 (190, 199) | 196 (191, 201) | 177 (168, 185) |
| **Statin cost, discounted (95% CI)** | 106 (104, 108) | 110 (107, 112) | 109 (107, 112) | 96 (94, 99) | 122 (120, 124) | 125 (123, 127) | 126 (124, 128) | 117 (114, 120) |
| **Statin Monitoring cost (95% CI)** | -1 (-1, -1) | -1 (-2, -1) | -2 (-2, -2) | 2 (1, 2) | -1 (-1, -1) | -1 (-1, -1) | -1 (-2, -1) | 2 (1, 2) |
| **Statin Monitoring cost, discounted (95% CI)** | -1 (-1, 0) | -1 (-1, -1) | -1 (-1, -1) | 1 (1, 1) | 0 (-1, 0) | -1 (-1, 0) | -1 (-1, -1) | 1 (1, 1) |
| **Incremental Total cost (£) (95% CI)** | 329 (247, 408) | 352 (264, 460) | 528 (383, 682) | 262 (105, 435) | 370 (275, 491) | 348 (262, 451) | 460 (331, 602) | 385 (192, 599) |
| **Incremental Total cost, discounted (£) (95% CI)** | 162 (123, 204) | 154 (112, 203) | 204 (135, 277) | 76 (-3, 168) | 185 (137, 245) | 165 (127, 212) | 201 (143, 266) | 149 (61, 251) |
| **ICER (£/QALY) (95% CI)** | 7427 (4615, 15088) | 4176 (2790, 6511) | 3182 (2132, 4700) | 2213 (-106, 5104) | 11778 (6155, 23971) | 5864 (4132, 9791) | 4951 (3218, 7804) | 5083 (2165, 10585) |

CI, confidence interval. CVD, cardiovascular disease. ICER, Incremental Cost-Effectiveness Ratio with costs and QALYs discounted at 3.5% per year. LDL, low density lipoprotein. QALY, quality-adjusted life years.

## Supplemental Table 5: Predicted discounted incremental health outcomes, costs and cost-effectiveness of standard statin treatment compared to no statin, in categories by sex, prior cardiovascular disease and pre-treatment LDL cholesterol level: base case and selected sensitivity analyses

| **Sex** | **Men** | | | | **Women** | | | |
| --- | --- | --- | --- | --- | --- | --- | --- | --- |
| **CVD history and LDL-C levels** | **Without CVD, LDL-C < 3.4** | **Without CVD, LDL-C 3.4-4.1** | **Without CVD, LDL-C ≥4.1** | **With prior CVD** | **Without CVD, LDL-C < 3.4** | **Without CVD, LDL-C 3.4-4.1** | **Without CVD, LDL-C ≥4.1** | **With prior CVD** |
| **Base case** |  |  |  |  |  |  |  |  |
| **Discounted Incremental QALYs** | 0.12 | 0.2 | 0.35 | 0.18 | 0.1 | 0.14 | 0.22 | 0.16 |
| **Discounted Incremental Primary care and Hospital admissions costs** | 100 | 75 | 302 | -358 | 60 | 29 | 93 | -170 |
| **Discounted Statin and Statin Monitoring costs** | 265 | 271 | 272 | 379 | 296 | 300 | 301 | 452 |
| **Discounted Incremental Total cost** | 365 | 347 | 574 | 21 | 355 | 328 | 394 | 282 |
| **ICER (£/QALY)** | 2945 | 1762 | 1655 | 116 | 3502 | 2276 | 1782 | 1725 |
| **Statin effects among participants >75 years old in RCTs** | | | | | | | | |
| **Discounted Incremental QALYs** | 0.08 | 0.14 | 0.24 | 0.14 | 0.07 | 0.10 | 0.15 | 0.12 |
| **Discounted Incremental Primary care and Hospital admissions costs** | 82 | 83 | 249 | -45 | 34 | 13 | 88 | -89 |
| **Discounted Statin and Statin Monitoring costs** | 265 | 272 | 272 | 376 | 295 | 300 | 300 | 449 |
| **Discounted Incremental Total cost** | 347 | 354 | 520 | 332 | 329 | 313 | 388 | 360 |
| **ICER (£/QALY)** | 4116 | 2603 | 2154 | 2311 | 5050 | 3285 | 2587 | 3080 |
| **Statin effects among participants >75 years old without cardiovascular disease at entry in RCTs** | | | | | | | | |
| **Discounted Incremental QALYs** | 0.05 | 0.07 | 0.13 | NA | 0.03 | 0.05 | 0.07 | NA |
| **Discounted Incremental Primary care and Hospital admissions costs** | -141 | -241 | -320 | NA | -144 | -210 | -283 | NA |
| **Discounted Statin and Statin Monitoring costs** | 263 | 270 | 268 | NA | 294 | 297 | 298 | NA |
| **Discounted Incremental Total cost** | 122 | 29 | -52 | NA | 150 | 89 | 16 | NA |
| **ICER (£/QALY)** | 2602 | 399 | -398 | NA | 4385 | 1856 | 218 | NA |
| **Lower relative risk reduction of vascular death of 7% (instead of 12%) per 1mmol/L LDL-C reduction with statin therapy** | | | | | | | | |
| **Discounted Incremental QALYs** | 0.10 | 0.17 | 0.30 | 0.14 | 0.08 | 0.12 | 0.19 | 0.13 |
| **Discounted Incremental Primary care and Hospital admissions costs** | -12 | -78 | 30 | -630 | -26 | -72 | -70 | -400 |
| **Discounted Statin and Statin Monitoring costs** | 265 | 271 | 270 | 377 | 295 | 299 | 300 | 450 |
| **Discounted Incremental Total cost** | 253 | 193 | 300 | -253 | 269 | 227 | 230 | 51 |
| **ICER (£/QALY)** | 2416 | 1152 | 1010 | -1781 | 3183 | 1877 | 1235 | 386 |
| **Results with 1.5% discount rate (instead of 3.5%) for outcomes and costs** | | | | | | | | |
| **Discounted Incremental QALYs** | 0.18 | 0.30 | 0.51 | 0.25 | 0.16 | 0.23 | 0.35 | 0.25 |
| **Discounted Incremental Primary care and Hospital admissions costs** | 287 | 330 | 826 | -133 | 193 | 178 | 363 | 64 |
| **Discounted Statin and Statin Monitoring costs** | 312 | 321 | 321 | 443 | 360 | 366 | 367 | 553 |
| **Discounted Incremental Total cost** | 598 | 651 | 1146 | 310 | 552 | 543 | 730 | 617 |
| **ICER (£/QALY)** | 3239 | 2199 | 2240 | 1240 | 3407 | 2350 | 2081 | 2495 |

CVD, cardiovascular disease. ICER, Incremental Cost-Effectiveness Ratio with costs and QALYs discounted at 3.5% per year (unless otherwise specified). LDL, low density lipoprotein. QALY, quality-adjusted life years. NA, not applicable.

## Supplemental Figure 1: Probability of statin therapy being cost-effective in older people in scenario analyses with CVD reductions with statin therapy in people>75 years old informed from effects of statin therapy among participants >75 years old (Scenario 1) or >75 years old without CVD (Scenario 2) from Cholesterol Treatment Trialists’ collaborative meta-analysis


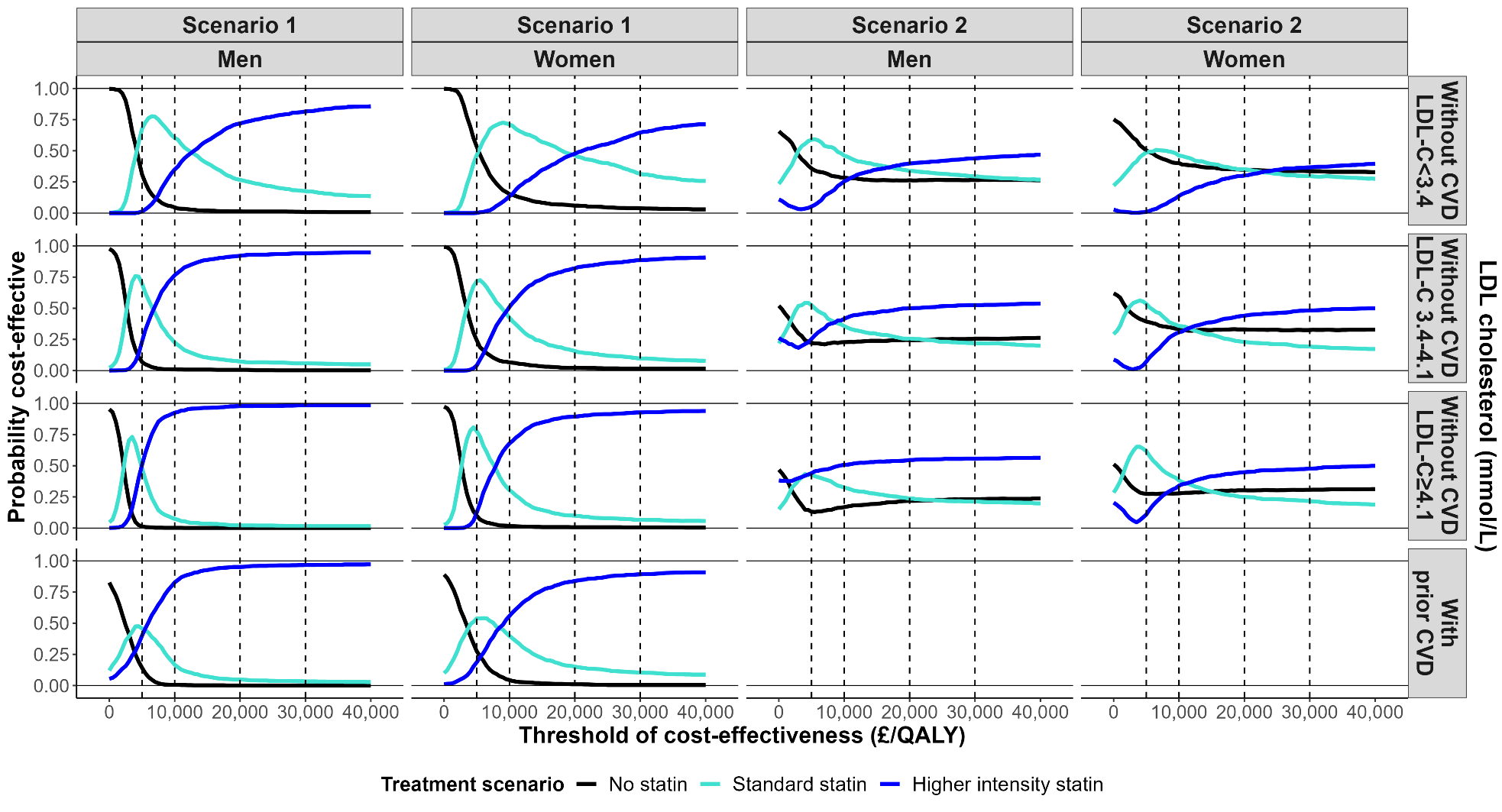


The probability that the treatment scenario provides the highest QALYs gain at the particular threshold of cost-effectiveness plotted. Statin effects up to age 75 as in base-case analysis; statin effect thereafter as per respective scenario analysis. CVD, cardiovascular disease. LDL, low density lipoprotein. QALY, quality-adjusted life years.

## Supplemental methods

### The CVD micro-simulation model

The model(2) was developed using the individual participant data of 16 large randomised clinical trials comparing statin versus control, and calibrated using the UK Biobank study’s(3) individual participant data. The model employs a broad range of patient socio-demographic characteristics (age, sex, ethnicity, physical activity, diet quality, quintile of socio-economic deprivation, body mass index (BMI), smoking status, blood pressure, serum lipid and creatinine levels, treated hypertension, and histories of CVD, diabetes (or HbA1c level for those without diabetes), cancer or mental illness) to project annually the first occurrence of four major CVD events: myocardial infarction, stroke, coronary revascularisation and vascular death, and three non-vascular events: incident diabetes, incident cancer and non-vascular death. Participant characteristics, disease histories and incident events determined health-related quality of life(2) and primary care and hospital admission costs(4) in each year of the model. The model was validated in categories of participants in UK Biobank and, separately, the Whitehall II study, and against national mortality, cancer incidence rates, and other published data. (2)

### Identifying participants 70 years and older

1. **UK Biobank**

Between 2006 and 2010, the UK Biobank study(3) recruited more than 500,000 40-70 years old men and women across the UK and followed them in resurveys and routine health records thereafter. At recruitment into the UK Biobank, given the general aim to recruit 40-69 year olds, only 2400 people aged 70-73 were recruited. By the time of the three further re-assessments of some UK Biobank participants (e.g. a random participant sample to assess variation in continuous biomarkers; re-assessment to collect baseline information for participants undergoing imaging assessments), some UK Biobank participants had reached age 70 years. Therefore, individuals aged ≥70 years at any UK Biobank assessment attendance from all four (re-)surveys were included from the earliest such attendance. A small number of participants with end stage kidney disease were excluded (see **supplemental methods table 1** for the details of inclusion).

**Supplemental methods table 1: distribution of included participants from all four UK Biobank surveys**

|  | **N** | **Data collection date** | **Age (years** | **Age>=70 years** | **Included*** |
| --- | --- | --- | --- | --- | --- |
| 1^st^ attendance (entry) | 502459 | 2006-03-13 to 2010-10-01 | 37-73 | 2423 | 2419 |
| 2^nd^ attendance | 20343 | 2009-12-12 to 2013-06-07 | 43-78 | 2716 | 2630 |
| 3^rd^ attendance | 56012 | 2014-04-30 to 2020-03-05 | 44-83 | 16896 | 12747 |
| 4^th^ attendance | 5305 | 2019-05-22 to 2020-03-05 | 49-83 | 1486 | 325 |

*Participants ≥70 years old with end stage kidney disease and those who were included from a previous attendance were excluded.

Definitions and specifications are the same as data preparation in the analyses of the main UK Biobank cohort data that was published previously. In general, analytical variables were coded using the UK Biobank nurse interview and linked electronic health records. Participants' characteristics at baseline or reassessments were derived based on data from interviews and linked records. Follow-up events, such as myocardial infarction (MI), stroke, coronary revascularization (CRV), incident diabetes, incident cancer, and mortality, were derived from linked death, hospital inpatient, cancer, and primary care (GP) records. Disease variables were coded referring to the International Classification of Diseases (ICD) 10 and 9 systems, UK Biobank cancer codes, UK Biobank non-cancer codes, and other UK Biobank algorithms. Procedure variables were coded referring to the Office of Population Censuses and Surveys (OPCS) 3 and 4 systems and UK Biobank operation codes. For more details, please refer to our earlier study’s supplemental table S1, and supplemental methods 1(2).

The end of follow-up date for these participants is the earliest from date of death, date of loss of follow-up, or 29/02/2020 (i.e. predating covid pandemic in UK). The average duration of follow-up is 3.5 years for the included individuals. The numbers of events during follow-up are summarised in **Supplemental methods table 2**.

**Supplemental methods table 2: Number of participants experiencing events during follow-up**

| **Cohort** | **Myocardial infarction** | **Stroke** | **Coronary revascularisation** | **Incident Cancer** | **Incident Diabetes** | **Vascular death** | **Nonvascular death** |
| --- | --- | --- | --- | --- | --- | --- | --- |
| **Without prior CVD** | 165 | 176 | 172 | 789 | 159 | 64 | 406 |
| **With prior CVD** | 67 | 92 | 136 | 285 | 99 | 75 | 152 |
| **Total** | 232 | 268 | 308 | 1074 | 258 | 139 | 558 |

CVD, cardiovascular disease.

1. **Whitehall II**

Whitehall II is a cohort study(5) among 10,308 participants aged 35-55 at entry, recruited from the British Civil Service in 1985, with periodic re-surveys of participants (called phases in Whitehall II). The Whitehall II study data includes Phases 1-9 and 11 at present. Due to attrition/no response and deaths, the numbers of participants declined from 10,308 in Phase 1 (1985-1988) to 6,308 in Phase 11 (2012-2014). We used participants from Phase 9 onwards for this external validation.

During odd-number phases, more detailed data was collected and biomarkers were measured. Routine healthcare data were linked for selected clinical events and mortality for participants. Electronic death records, NHS hospital records and cancer registry entries were linked for Whitehall II participants and used to identify events during follow-up. However, this data were made available until particular time points for different types of events. For example, the data for strokes was available until about Phase 9, while the data for MI, cancer and death were available over longer time period. We used the linked clinical events and mortality data to identify events during follow-up, but also used questionnaire data to complement data for some events (see **Supplemental methods table 3** for more details). For incident CRVs and incident diabetes, only partial questionnaire data were available (for CRV including only coronary artery bypass graft surgery, date of incident diabetes not systematically recorded); linked healthcare data were not available. Therefore, we did not validate the model for these endpoint.

**Supplemental methods table 3: Identifying incident events for the older Whitehall II cohort**

|  | **Linked electronic health data** | **Whitehall II questionnaire data** |
| --- | --- | --- |
| Myocardial infarction (MI) | Electronic hospital and death records of “MI (HES/death)” until the mid of 2019 | Not used |
| Stroke | Electronic hospital and death records of “All stroke (HES/death)” until the mid of 2019 |  |
| Cancer | Electronic cancer registry “All malignant cancer” with follow-up until March 2015 used. | Variables indicating self-reported “ever told cancer” were used to identify further baseline cancer as a complement. As only told years were available, 30^th^ June of the told year was assumed as the told date. Told cancers with an unknown year, mostly from Phase 1, were assumed to occur on 1^st^ January 1985, the year when Whitehall II project started. |
| Vascular death (VD) | Electronic death certificate data (until February 2021) were used; CVD death category; cut at 28 February 2020 to avoid the impact of covid19 | Electronic death certificate data (until February 2021) were used; CVD death category; cut at 28 February 2020 to avoid the impact of covid19 |
| Nonvascular death (NVD) | Electronic death certificate data (until February 2021) were used; non-CVD death category; cut at 28 February 2020 to avoid the impact of covid19 | Electronic death certificate data (until February 2021) were used; non-CVD death category; cut at 28 February 2020 to avoid the impact of covid19 |

CVD, cardiovascular disease. HES, hospital episodes statistic.

For the participants in Phase 9, deaths have the longest follow-up periods, with an average of 10.9 years, followed by MI with an average of 9.4 years and stroke 9.3 years. The follow-up periods of cancers are shorter, with an average of 6.1 years. The numbers of follow-up events for the older cohort are summarised in Supplemental methods table 4.

**Supplemental methods table 4: Number of Whitehall II participants 70 years and older at Phase 9 experiencing events**

|  | **Myocardial infarction (MI)** | **Stroke** | **Incident Cancer** | **Vascular death (VD)** | **Nonvascular death (NVD)** |
| --- | --- | --- | --- | --- | --- |
| **Without history of CVD (n=1247)** | 46 | 56 | 170 | 75 | 290 |
| **With history of prior CVD (n=754)** | 84 | 63 | 90 | 93 | 210 |
| **Total (N=2001)** | 130 | 119 | 260 | 168 | 500 |

CVD, cardiovascular disease.

### Handling missing data

1. **UK Biobank**

We initially created a panel dataset for all individuals who attended as least one of the three follow-up surveys of UK Biobank, so in this dataset an individual has a baseline value and 1-3 follow-up values for each characteristic. The summary of missingness and imputation methods is presented in **Supplemental methods table 5**. In general, characteristics such as sex, ethnicity and Townsend score are invariant across time. A small numbers of missing smoking status were imputed using the Last observation carried forward (LOCF) method. The diet categorisation used the same method as previously, in which a small number of uncertain diet types due to missing values in consumption of some food types were combined with unhealthy diet. Physical activity levels are missing for all follow-up surveys, and they were imputed as the baseline levels.

We multiply imputed the missing biomarker values in a wide form (one individual one row and multiple measures for one characteristic) by chained equations using the Markov chain Monte Carlo (MCMC) simulation using a R package “mice”. All characteristics of analyses were included, plus a binary variables of statin use at survey administration. All characteristics with missing values at entry into UK Biobank have been imputed as specified in our previous analysis. 80 imputations and 40 maximum iterations were used in the multiple imputation and the means across all the imputations were used to impute the missing values.

LDL, HDL, creatinine and HbA1c values were not measured at second and third follow-up re-surveys, and therefore they were missing. They were not imputed in the wide-form multiple imputation. Instead, we implemented a long-form multiple imputation. In the long form, all multiple measures of a characteristic such as LDL were stacked in one variable, which had values from baseline and the first follow-up survey and therefore can be imputed. Additionally, durations from entry to the current attendance were used, so that the trend was taken into account. The long-form multiple imputation model specification and parameters were then identical to the wide-form multiple imputation.

**Supplemental methods table 5: Summary of missing data and imputation method in UK Biobank re-assessments**

|  | **Reassessment 1** | **Reassessment 2** | **Reassessment 3** | **Imputation method** |
| --- | --- | --- | --- | --- |
| **Baseline Characteristics** | **Numbers of missing values** | | |  |
| Age at recruitment | As baseline | As baseline | As baseline | NA |
| Age at re-assessment | 0 | 0 | 0 | NA |
| Sex | As baseline | As baseline | As baseline | NA |
| Ethnicity | As baseline | As baseline | As baseline | NA |
| Townsend score | As baseline | As baseline | As baseline | NA |
| Smoking | 60 | 518 | 38 | LOCF |
| BMI (kg/m^2^) | 46 | 1638 | 19 | Wide-form multiple imputation |
| Systolic blood pressure (mmHg) | 13 | 5952 | 266 | As above |
| diastolic blood pressure (mmHg) | 13 | 5952 | 266 | As above |
| CVD history | 0 | 0 | 0 | NA |
| Type 1 diabetes | 0 | 0 | 0 | NA |
| Hypertension treatment | 0 | 0 | 0 | NA |
| LDL (mmol/L) | 2508 | ALL | ALL | Wide-form multiple imputation for re-assessment 1; long-form multiple imputation for re-assessments 2&3 |
| HDL (mmol/L) | 4654 | ALL | ALL | As above |
| Creatinine (umol/L) | 2502 | ALL | ALL | As above |
| HbA1c (mmol/mol) | 6077 | ALL | ALL | As above |
| Physical activity | ALL | ALL | ALL | As baseline |
| Severe mental illness | 0 | 0 | 0 | NA |
| Diet | 130 | 661 | 43 | Combined missing with unhealthy/ uncertain |

BMI, body mass index. CVD, cardiovascular disease. HbA1c, hemoglobin A1c. HDL, high density lipoprotein. LOCF, last observation carried forward. LDL, low density lipoprotein. NA, not applicable.

1. **Whitehall II**

We focused on Whitehall II Phase 9 data. Referring to the specification of model input characteristics, there are small numbers of missing values for ethnicity, Townsend score, smoking status and disease histories, moderate numbers of missing values for biomarkers (8%-15%), and data was not available for prior Type 1 diabetes, severe mental illness and diet quality. We assigned the missing values to the most common categories in UK Biobank older cohort, referred to the available values in the last phase, or used multiple imputation by chain equations to address missing data (**Supplemental methods table 6**). The parameter specification of multiple imputation was the same as for the older UK Biobank cohort, and data about individuals across all ages and data from Phase 5, Phase 7 and Phase 11 were additionally included to provide information for multiple imputation.

Particularly, Whitehall II only provides two categories of ethnicity, White and non-White. We made the assumption that all non-White were South Asians, the second largest ethnicity group in England. The Whitehall II’s physical activity categories include the moderate level and the low level (high-level physical activity category was possibly combined with the moderate level in Whitehall II). We assumed that participants did not have Type 1 diabetes, nor sever mental illness and all had “healthy” diet, the most common category in UK Biobank.

**Supplemental methods table 6: Summary of missing data and imputation method in Whitehall II**

| **Variables** | **Numbers of missing values** | **Methods** |
| --- | --- | --- |
| Age at attendance | 0 | Age at phase attendance; no missing |
| Sex | 0 | No missing |
| Ethnicity | 5 | Missing values assigned to “White”;  Non-white assigned to “South Asian” |
| Townsend score | 100 | Multiply imputed |
| Smoking | 145 | Refer to status in last phase; otherwise assigned to the most common category, i.e. non-smoker |
| BMI | 248 | Multiply imputed |
| CVD history | 93 | Myocardial infarction (MI), stroke and other coronary heart diseases (total angina) used clinical follow-up prevalence data, complemented by questionnaire data.  Peripheral artery disease (PAD) used only questionnaire data referring to the algorithm used in Health Survey for England 2017. |
| Prior cancer | 2 | Based on Clinical follow-up data, complemented by questionnaire data “ever told cancer” (available at Phase 1, 4 & 5). Only years of being told are available (for Phase 4 & 5), so assume the day as 30^th^ June of that year; other unknown dates (mostly from Phase 1) were assumed to be “1^st^ January 1985” (WH2 starting year). |
| Prior diabetes | 3 | Questionnaire data only “self-reported diabetes”; assume diagnosis date of diabetes before Phase 1 to be “1^st^ January 1985” |
| Type 1 diabetes | 0 | Coding was based on self-reported other long-term illness. Participants who did not report any long-term illness were assumed no prior such illness |
| Treated hypertension | 5 | Minor missing, assigned to “no treated hypertension” |
| LDL-C (mmol/L) | 305 | Multiply imputed |
| HDL-C (mmol/L) | 298 | Multiply imputed |
| Creatinine (umol/L) | 298 | Creatinine was not measured at Phase 7 but measured at phase 9.  Missing values multiply imputed. |
| HbA1c (mmol/mol) | 301 | Multiply imputed |
| Systolic blood pressure (mmHg) | 240 | Multiply imputed |
| diastolic blood pressure (mmHg) | 240 | Multiply imputed |
| Physical activity | 65 | Only three levels: low, moderate & missing (missing is a separate category, corresponding to the UK Biobank data) |
| Prior severe mental illness | 0 | Coding was based on self-reported other long-term illness. Participants who did not report any long-term illness were assumed no prior such illness |
| Healthy diet | 443 | All assigned to unhealthy diet, corresponding to the method used in the UK Biobank data |

BMI, body mass index. CVD, cardiovascular disease. HbA1c, hemoglobin A1c. HDL, high density lipoprotein. LDL, low density lipoprotein.

### Derivation of pre-treatment LDL cholesterol levels for statin-treated UK Biobank and WHITEHALL II participants

For UK Biobank participants, we adjusted upwards the LDL cholesterol levels of participants who were on statin treatment at entry into UK Biobank to derive “pre-treatment” LDL cholesterol levels using the potency of their statin regimen (see Supplemental Table 1). Statin dosage information was not collected at UK Biobank baseline interview but for participants with linked primary care prescription records both type of statin and dosage were available. However, more than half of the UK Biobank participants did not have linked primary care data, and, therefore, no linked primary care prescription records. Additionally, there were some discrepancies between reported statin use at UK Biobank recruitment and available statin prescription records; for such cases a report of statin use in either source was accepted and, if more than one source available, the more intensive regimen was used.

The statin treatment reports with an exact drug type and dosage were directly associated with the proportional LDL-C reductions according to Supplemental Table 1. The statin treatment with unknown dosage were assumed to derive the average proportional LDL cholesterol reduction according to weighted frequency of statin regimens within the primary or secondary prevention populations with known statin regimens.

Due to a complete lack of information about the exact drug type and dosage for statin treatment in Whitehall II Phase 9 participants, proportional LDL-C reductions among participants without prior CVD and with prior CVD were assumed to be 35% and 45%, respectively, informed by the corresponding average values in the UK Biobank participants.

The **pre-treatment LDL cholesterol (LDL-C)** of statin-treated participants was calculated as:

LDL-C _pre-treatment_ = LDL-C _entry_ / (1 - %LDL-C reduction on statin treatment)

### Integrating treatment effects of statin therapy in the CVD model

Statin treatment effects based on the rate ratios (RR) per 1mmol/L reduction in LDL cholesterol, as reported by Cholesterol Treatment Trialists’ Collaborative meta-analysis(6), were used for effects on myocardial infarction, stroke, coronary revascularisation and vascular death and further meta-analyses informed effects of statin therapy on incident diabetes in the model(7, 8) (see Table 1). In the base-case analysis, it was assumed that statins do not affect cancer incidence and non-vascular death.

The transition probabilities (tp) of events in the absence of statin treatment in the model in each cycle are calculated as:

**tp(t_u_) = 1 – exp[H(t-u) – H(t)]**, where

u is the length of the cycle (i.e. 1 year), H(t-u) and H(t) are the cumulative hazards at time t-u and t, respectively.

The treatment effects of statin (tx) are calculated as: **tx = exp[ALR * ln(RR)]**, where RR is the rate ratio per 1 mmol/L LDL cholesterol reduction with statin (see Table 1) and ALR is the absolute LDL cholesterol reduction with the statin therapy, which is product of pre-treatment LDL cholesterol level and the proportional reduction in LDL cholesterol with corresponding statin regimen (see Supplemental Table 1).

The transition probabilities for events with statin treatment (tp_tx_) in each cycle of the model is calculated as:

**tp_tx_(t_u_) = 1 – exp[H(t-u) – H(t)]^tx^**

The excess rates on myopathy and rhabdomyolysis of statin treatment (see Table 1) were applied as constant annual rate each year on statin treatment in the model.

In the CVD microsimulation model, the first occurrences of MI, stroke and coronary revascularisation (CRV) were modelled followed by vascular death. The statin treatment effects, estimated using intention-to-treat analyses for times to first occurrences of these endpoints across the randomised studies in the Cholesterol Treatment Trialists’ Collaborative meta-analysis, informed the treatment effects in the model. Therefore, we considered whether we may double count some treatment effects by simulating the effects on cardiovascular events concurrently (e.g. modelling statin impact on vascular death both through its effects on MI, stroke, CRV as well as its direct effect on risk of VD). We therefore internally validated the differences in predicted cumulative incidences of these endpoints due to statin therapy against the differences between observed censoring-adjusted cumulative incidences of these endpoints in the Cholesterol Treatment Trialists’ Collaborative database (using the initial CVD model developed in this database(3)). The predicted effects were consistently within the 95% confidence intervals of the observed differences across the 5 years of follow-up in the trials. Nevertheless, to explore sensitivity of finding to somewhat smaller effect on cardiovascular death, in a sensitivity analysis the effect of statin therapy on vascular death was set at RR 0.93 (instead of the base case RR of 0.88 per 1mmol/L LDL cholesterol reduction), corresponding to the estimated effect of allocation to statin per 1mmol/L LDL cholesterol reduction with time-varying adjustment for first post randomisation occurrences of MI, stroke and CRV).

### Specification of sensitivity and scenario analyses

**Supplemental methods table 7: Sensitivity analyses parameterisation in the model**

| **Scenario** | **Parameters** |
| --- | --- |
| **Base-case analysis (see Table 1)** | - Overall effect of statin therapy on cardiovascular events (per 1 mmol/L LDL cholesterol reduction)(6):   MI: Major coronary event Rate Ratio (RR) 0.76 (95% CI 0.73, 0.79)  Stroke RR 0.84 (95% CI 0.80, 0.89)  CRV RR 0.75 (95% CI 0.73, 0.78)  VD RR 0.88 (95% CI 0.85, 0.91)   - Statin does not have any effect on cancer - Compliance with statin therapy 100% - No disutility of daily statin - 3.5% discount rate for costs and outcomes - All healthcare costs included - Cost of statin therapy as per NHS Drug Tariff December 2021 |
| **Statin treatment effects on cardiovascular events after age 75 years corresponding to statin effects among participants >75 years of age in Cholesterol Treatment Trialists’ Collaborative meta-analysis (Scenario 1)** | The applied effects based on Cholesterol Treatment Trialists’ individual participant data meta-analysis, reporting effects only among participants >75 years of age at randomisation (per 1 mmol/L LDL cholesterol reduction) (6) were:  MI: Major coronary event RR 0.82 (99% CI 0.70, 0.96)  Stroke RR 0.89 (99% CI 0.71, 1.10)  CRV RR 1.02 (99% CI 0.75, 1.40)  VD RR 0.95 (99% CI 0.83, 1.07) |
| **Statin treatment effects on cardiovascular events after age 75 years corresponding to statin effects among participants >75 years of age and without CVD at randomisation in Cholesterol Treatment Trialists’ Collaborative meta-analysis (Scenario 2)** | The applied effects based on Cholesterol Treatment Trialists’ individual participant data meta-analysis: effects only among participants >75 years of age and without CVD at randomisation (per 1 mmol/L LDL cholesterol reduction) (6) (personal communication):  MI: Major coronary event RR 0.87 (99% CI 0.65, 1.15)  Stroke RR 0.88 (99% CI 0.59, 1.30)  CRV RR 0.93 (99% CI 0.50, 1.74)  VD RR 1.04 (99CI 0.76, 1.43) |
| **Smaller statin treatment effect on vascular death** | Relative reduction in vascular death with statin RR 0.93 per 1 mmol/L LDL cholesterol reduction). |
| **Vary statin treatment effect on cancer incidence** | RR of 0.96 or 1.05, respectively, applied for incident cancer with statin therapy based on 95% confidence interval of the Cholesterol Treatment Trialists’ individual participant data meta-analysis reporting RR of 1.00 (95% CI 0.96, 1.05).(9) |
| **Real-world compliance with statin therapy** | Using observed statin discontinuation and restarting rates for the first discontinuation and first restarting,(10) the derived probabilities of complying with statin therapy (Supplemental methods table 8) were applied to each individual in the respective years in model simulation. Both statin effects and costs were discontinued with no statin use. |
| **Quality of life disutilities of daily statin pill** | 0.001, 0.002 or 0.005 QALYs were deducted in each model year. (11) |
| **Higher risk of nonvascular death and lower general QoL** | Includes three scenarios across all alternatives: 1) doubling individual’s risk of nonvascular death; 2) reducing individual’s QoL by 0.1 each year; 3) both doubling NVD risk and reducing QoL by 0.1 |
| **Discount rates for costs and outcomes of 1.5%** | Annual discount rates to 1.5% were used for costs and QALYs (instead of the 3.5% base-case rates) |
| **Include healthcare costs only for CVD and incident diabetes** | Healthcare costs associated with CVD and incident diabetes only included (ie, unrelated healthcare costs were excluded). |
| **Increased cost of statin therapy** | The base-case costs of statin therapy increased 1.5, 2 or 5 times. |

RR, rate ratio. MI, myocardial infarction. CRV, coronary revascularisation. QALY, quality-adjusted life years. QoL, quality of life. RR, relative risk. VD, vascular death.

**Supplemental methods table 8: Probabilities for first discontinuation and first restarting of statin treatment and the derived probabilities of compliance with statin therapy over the first 10 years**

|  | Cumulative probability (%)(10) | | On statin treatment (%) | |
| --- | --- | --- | --- | --- |
| Year | Discontinuation | Restarting | On | Off |
| 1 | 30% | 50% | 70% | 30% |
| 2 | 38% | 59% | 77% | 23% |
| 3 | 43% | 64% | 79% | 21% |
| 4 | 47% | 68% | 80% | 20% |
| 5 | 50% | 70% | 81% | 19% |
| 6 | 52% | 72% | 81% | 19% |
| 7 | 54% | 74% | 82% | 18% |
| 8 | 56% | 76% | 82% | 18% |
| 9 | 58% | 77% | 83% | 17% |
| 10 | 60% | 79% | 83% | 17% |

The first two columns present cumulative probabilities for the first discontinuation and first restarting of statin treatment(10), followed by the derived compliance with statin treatment in first 10 years of treatment.

## Supplemental material references

1. Law MR, Wald NJ, Rudnicka AR. Quantifying effect of statins on low density lipoprotein cholesterol, ischaemic heart disease, and stroke: systematic review and meta-analysis. BMJ. 2003;326(7404):1423.

2. Wu R, Williams C, Zhou J, Schlackow I, Emberson J, Reith C, et al. Long-term cardiovascular risks and statin treatment impact on socioeconomic inequalities: microsimulation model. Brit J Gen Pract. 2023:BJGP.2023.0198.

3. Sudlow C, Gallacher J, Allen N, Beral V, Burton P, Danesh J, et al. UK Biobank: an open access resource for identifying the causes of a wide range of complex diseases of middle and old age. PLoS Med. 2015;12(3):e1001779.

4. Zhou J, Wu R, Williams C, Emberson J, Reith C, Keech A, et al. Prediction Models for Individual-Level Healthcare Costs Associated with Cardiovascular Events in the UK. Pharmacoeconomics. 2023;41(5):547-59.

5. Marmot M, Brunner E. Cohort Profile: the Whitehall II study. Int J Epidemiol. 2005;34(2):251-6.

6. Cholesterol Treatment Trialists' Collaboration. Efficacy and safety of statin therapy in older people: a meta-analysis of individual participant data from 28 randomised controlled trials. Lancet. 2019;393(10170):407-15.

7. Sattar N, Preiss D, Murray HM, Welsh P, Buckley BM, de Craen AJ, et al. Statins and risk of incident diabetes: a collaborative meta-analysis of randomised statin trials. Lancet. 2010;375(9716):735-42.

8. Preiss D, Seshasai SR, Welsh P, Murphy SA, Ho JE, Waters DD, et al. Risk of incident diabetes with intensive-dose compared with moderate-dose statin therapy: a meta-analysis. JAMA. 2011;305(24):2556-64.

9. Cholesterol Treatment Trialists' (CTT) Collaboration. Lack of effect of lowering LDL cholesterol on cancer: meta-analysis of individual data from 175,000 people in 27 randomised trials of statin therapy. PLoS One. 2012;7(1):e29849.

10. Pate A, Elliott RA, Gkountouras G, Thompson A, Emsley R, van Staa T. The impact of statin discontinuation and restarting rates on the optimal time to initiate statins and on the number of cardiovascular events prevented. Pharmacoepidemiol Drug Saf. 2020;29(6):644-52.

11. Hutchins R, Viera AJ, Sheridan SL, Pignone MP. Quantifying the utility of taking pills for cardiovascular prevention. Circ Cardiovasc Qual Outcomes. 2015;8(2):155-63.
